# Automated approach for the evaluation of glutathione-S-transferase P1-1 inhibition by organometallic anticancer compounds

**DOI:** 10.1080/14756366.2022.2073443

**Published:** 2022-05-29

**Authors:** Sarah A. P. Pereira, A. Catarina Baptista L, Lorenzo Biancalana, Fabio Marchetti, Paul J. Dyson, M. Lúcia M. F. S. Saraiva

**Affiliations:** a LAQV, REQUIMTE, Departamento de Ciências Químicas, Faculdade de Farmácia, Universidade do Porto, Porto, Portugal; b Dipartimento di Chimica e Chimica Industriale, Università di Pisa, Pisa, Italy; c Institut des Sciences et Ingénierie Chimiques, École Polytechnique Fédérale de Lausanne (EPFL), Lausanne, Switzerland

**Keywords:** Sequential injection analysis (SIA), glutathione S-transferase P1-1, enzyme inhibition assays, anticancer metal complexes

## Abstract

A novel automated method based on sequential injection analysis (SIA), a non-segmented flow injection technique, was developed to evaluate glutathione S-transferase P1-1 (GST P1-1) activity in the presence of organometallic complexes with putative anticancer activity. The assay is based on the reaction of L-glutathione (GSH) and 1-chloro-2,4-dinitrobenzene (CDNB) in the presence of GST P1-1 to afford the GS-DNB conjugate and the reaction may be monitored by an increase in absorbance at 340 nm. A series of ruthenium, iron, osmium and iridium complexes were evaluated as GST P1-1 inhibitors by evaluating their half-maximal inhibitory concentration (IC_50_). An iridium compound displays the lowest IC_50_ value of 6.7 ± 0.7 µM and an iron compound displays the highest IC_50_ value of 275 ± 9 µM. The SIA method is simple to use, robust, reliable, and efficient and uses fewer reagents than batch methods and each analysis takes only 5 minutes.

## Graphical Abstract

**Figure:**
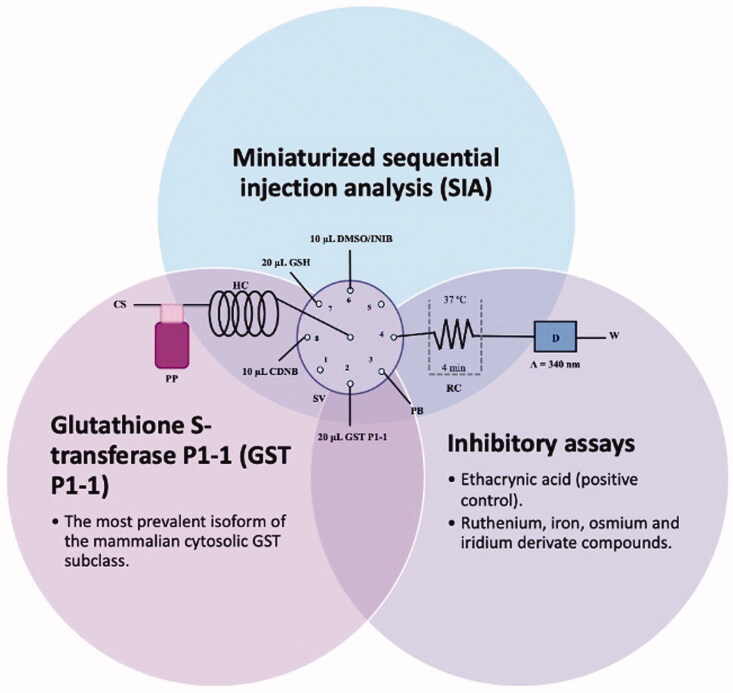

## 1. Introduction

Glutathione S-transferases (GSTs) are a superfamily broadly distributed in phase II metabolism enzymes that catalyse the conjugation of extensive diversity of reactive electrophiles to the nucleophilic sulphur atom of tripeptide glutathione (γ-L-glutamyl-L-cysteinyl glycine, GSH). After formed, the hydrophilic GSH conjugates are successfully removed from the cell, inducing the detoxification of the organism^1^^,^^2^. The greatest predominant isoform of the GST subclass in mammalian cytosolic is GST P1-1, and its overexpression can be directly correlated to carcinogenesis and chemotherapeutic drug resistance^3^^,^^4^. This isoform is overexpressed in human tumours such as ovarian, kidney, and breast carcinoma^5^^,^^6^, with its overexpression accelerating drug metabolism leading to a decrease in therapeutic efficacy^7^.

Several GST inhibition batch assays have been reported resorting to a different mode of detection, such as an electrochemical assay using a glassy carbon electrode with differential pulse voltammetry to evaluate GST kinetic parameters^8^, or an immunocytochemistry technique to evaluate the cellular reactivity of GSTπ^9^. With a higher level of mechanisation, a high-resolution screening (HRS) technique using two simultaneous enzyme affinity detection (EAD) systems for human GST P1-1 using reverse-phase high-performance liquid chromatography (HPLC). This system was first optimised and validated using a flow injection analysis (FIA) system and the optimised results were then used in HPLC mode^10^.

In this work, a sequential injection analysis (SIA) system was developed to assess GST P1-1 activity and evaluate several organometallic compounds with putative anticancer activity. SIA was chosen rather than FIA, as it is better suited to high-cost enzymes/reagents and complicated multi-step reactions since it is possible to use fewer volumes and present several reagents handling abilities^11^ and minimises some of the drawbacks of batch assays by ensuring effective control of the reaction conditions^12^, significantly impacting precision and accuracy^13^. In SIA, enzymatic activity is determined in the early stages of the reaction avoiding interference from low-affinity substrates. Compared to FIA, SIA is more versatile, with computer control mode, and the implementation of different analytical procedures without physical reconfiguration of the setting^14^.

SIA is an automatic approach that enables the performance of wet-chemistry procedures in a rapid, precise, and efficient manner. SIA systems have been broadly accomplished in the last decades for the application of enzyme-based assays aiming at the evaluation of enzyme activity, enzyme inhibition assays, and the determination of specific analytes.

The SIA method reported herein is based on the GST P1-1 catalysed reaction of 1-chloro-2,4-dinitrobenzene (CDNB) with reduced glutathione (GSH) which results in an increase in absorbance at 340 nm. Following validation of the assay using ethacrynic acid (EA), a benchmark GST P1-1 inhibitor^15^, a selection of organometallic iron, ruthenium, osmium, and iridium complexes, currently investigated for their anticancer activity, were tested to evaluate the inhibition capacity against GST P1-1 enzyme. Iron is attractive for developing metal-based drugs due to its bioavailability and the feasible redox chemistry in physiological media^16–18^. Recently, organometallic diiron compounds based on the {Fe_2_Cp_2_(CO)_x_} scaffold (*x* = 2 or 3) were shown to display selective cytotoxicity to certain cancer cells. Organoruthenium (half-sandwich) compounds have been extensively studied over the last two decades due to their promising anti-cancer properties^19^, with some even validated in vivo against cancers with a very poor prognosis^20^^,^^21^. Related osmium and iridium half-sandwich complexes have received far less attention than those of iron and ruthenium concerning their application in medicinal chemistry, but several promising results have been reported^22^^,^^23^. The conjugation of known enzyme inhibitors to metal-based drugs emerged as a prominent strategy to develop effective anticancer compounds^24^, with early examples corresponding to half-sandwich ruthenium complexes modified with EA^25^^,^^26^, and some of the organometallic compounds studied herein have pendant EA groups^25^^,^^27^.

## 2. Materials and methods

### 2.1. Reagents and solutions

Glutathione S-transferase P1-1 (GST P1-1); 1-Chloro-2,4-dinitrobenzene (CDNB), glutathione (GSH), and ethacrynic acid (EA) were purchased from Sigma. Dimethyl sulfoxide (DMSO) and ethanol were purchased from Merck and Fisher Chemicals, respectively. Ultrapure water obtained from the MILI-Q plus system with a specific conductivity of < 0.1 μS cm^− 1^ was used to prepare all the solutions.

CDNB and GSH were daily prepared in ethanol and phosphate buffer 0.1 mol L^−1^ pH 6.5 at 44 mM and 12 mM, respectively. GST P1 was reconstituted from a solution comprising 50 mM Tris-HCl at pH 7.5 with 50 mM of sodium chloride (NaCl), and 1 mM of 1,4-dithiothreitol (DTT), 5 mM of ethylenediaminetetraacetic acid (EDTA), and 50% of glycerol. The GST P1 solution (0.2 µM) used in the assays was incubated in an ice bath during the procedure. A 0.1 mol L^−1^ phosphate buffer solution (pH 6.5) was applied as a carrier solution for the SIA method. Compounds **2a–d**^28^**, 3a**^29^**, 4a–d**^30^^,^^31^**, 5a–f**^32–34^**, 6a–b**^35^^,^^36^**, 7a–e**^37–40^ were prepared in agreement to literature methods and were dissolved in DMSO.

### 2.2. Analytical apparatus

**Figure 1.**
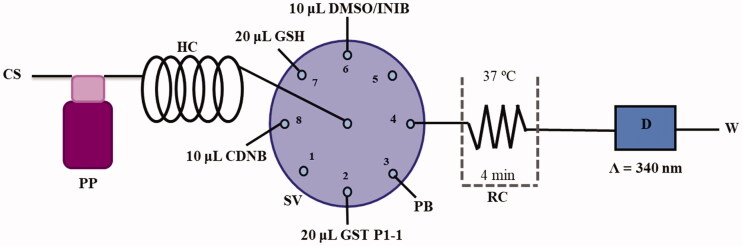
Illustration of the SIA manifold used. CS – Carrier solution; HC – holding coil; PP – peristaltic pump; SV – Selection valve; DMSO/INIB – dimethylsulfoxide/inhibitor; CDNB- 1-Chloro-2,4-dinitrobenzene; GSH – Glutathione reduced; GST P1-1 – Glutathione S-transferase P1-1; PB – Phosphate buffer; RC – Reaction coil; D – Detector and W – Waste.

The SIA system is represented in Figure 1 and consists of a selection valve Crison^®^ module with 8 ports and a peristaltic pump Gilson^®^ Mini plus 3 sets with a pumping tube of polyvinyl chloride with 1.30 mm i.d. All the system components are connected by Teflon tubes of 0.8 mm in diameter. A reactor coil of 50 cm in length was immersed inside the thermostatic bath to maintain the mixture at 37 °C.

Measurements were performed using a Jenway^®^ 6300 spectrophotometer detector, incorporating an 80 µL flow cell (Hellma Analytics^®^), connected to the reactor coil, with 10 mm of optical path length. The absorption wavelength was fixed at 340 nm. Microsoft QuickBasic 4.5 software was used to control the flow system.

### 2.3. Sequential injection analysis procedure

The half-maximal inhibitory concentration (IC_50_) determination of GST P1-1 activity of the compounds was performed using the SIA system as follows. Before starting the analytical cycle, all the system tubes were filled with the carrier solution (phosphate buffer at pH 6.5). Then the tubes from positions 2, 3, 6, 7, and 8 were filled with GST P1-1, phosphate buffer pH 6.5, inhibitor, GSH, and CDNB, respectively. Afterward, the analytical cycle, presented in Table 1, was carried out by the aspiration of 10 µL of CDNB, 10 µL of DMSO/inhibitor, 20 µL of GST P1-1, and 20 µL of GSH (steps 1–4). Then, the aliquots were propelled to the reaction coil (RC) by flow reversal (step 5) and the flow was stopped inside the RC for 4 min to promote the reaction product formation (step 6). After this stop period, the reaction product was propelled to the detection cell (step 7), and the analytical signal was recorded. All the determinations were carried out at 37 °C and each assay was performed in triplicate.

**Table 1.** Analytical cycle used to perform the GST P1-1 inhibition assays.

Step	Position valve	Reagent	Volume (µL)	Time (s)	Flow rate (mL min ^-1^)	Action
1	8	CDNB	10	1.2	0.5	Aspiration of CDNB
2	6	DMSO/ inhibitor	10	1.2	0.5	Aspiration of DMSO/inhibitor
3	2	GST P1-1	20	2.4	0.5	Aspiration of GST P1-1
4	7	GSH	20	2.4	0.5	Aspiration of GSH
5	4	Mixture	333	20	1	Propulsion to the reactor coil
6	4		–	240	0	Stop period in reactor coil
7	4	Mixture	2000	60	2	Propulsion to the detector

### 2.4. Batch procedure

**Figure 2.**
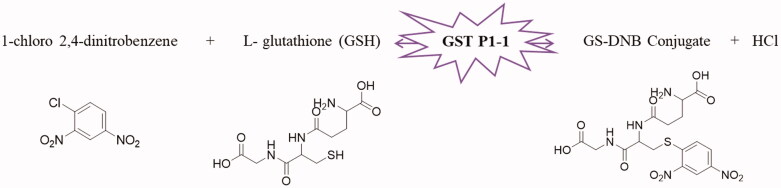
GST P1-1 enzymatic reaction.

To evaluate the enzymatic reaction, a concentration of GST P1 of 20 nM was spectrophotometrically determined at 340 nm by monitoring the reaction of CDNB (1 mM) with GSH (2 mM) (Figure 2) over 8 min in 0.1 M potassium phosphate buffer at pH 6.5 based on a previously reported protocol^41^. All the assays were performed at around 37 °C and in triplicate. The IC_50_ values were acquired using GraphPad Prism 7 software.

### 2.5. Data analysis

The evaluation of the inhibition curves was performed using GraphPad Prism 7 software using the equation defined by [Inhibitor] vs. normalised responsible–variable slope, where *X* values should be concentrations, not transformed to logarithms and the *Y* values of the curve were go from 100 down to 0. This model corresponds to the equation Y=1001+(IC50X)^Hillslope.

To obtain the normalised activities for each inhibitor concentration, we assume that 100% is the maximum activity of the reaction without the presence of an inhibitor. 100% is equal to 1, so each percentage of inhibition is converted into a normalised activity (a number between 0 and 1, being 0 and 1 equal to 0% and 100%).

## 3. Results and discussion

### 3.1. Optimisation of the SIA methodology

The first stage of the SIA method development comprised the evaluation of the physical configuration and the chemical factors that affect the reaction. For this, it was used the univariate approach was where each parameter is improved while the others are maintained constant. The main parameters studied include the reaction time, the reagents aliquots volume, their aspiration order, and the temperature. Table 2 lists these optimised parameters with the studied range and the chosen values.

**Table 2.** SIA system optimisation.

Condition	Range	Selected value
Stop period (min)	0–5	4
Aspiration order	GSH – DMSO/inhibitor – GST P1-1 – CDNB GSH – GST P1-1 – DMSO/inhibitor – CDNB CDNB – DMSO/inhibitor – GST P1-1 – GSH CDNB – GST P1-1- DMSO/inhibitor – GSH	CDNB – DMSO/inhibitor – GST P1-1 – GSH
Temperature (°C)	25–37	37
GST volume (µL)	10–25	20
GSH volume (µL)	15–25	20

**Figure 3.**
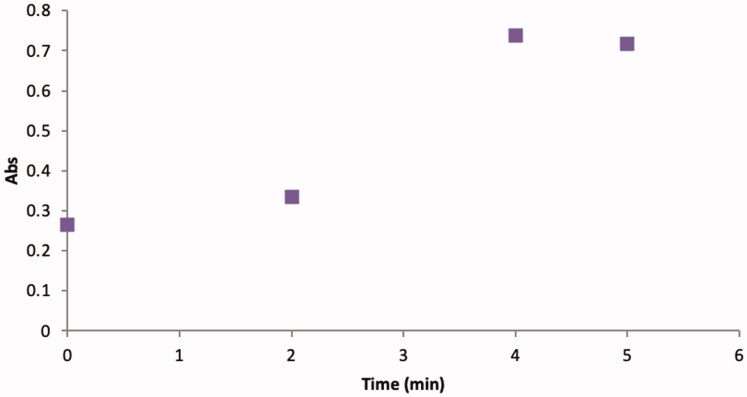
Optimisation of the GST P1-1 reaction time.

GST P1 activity was evaluated using a flow injection methodology, with a stopped-flow period at the reaction coil, enabling the GS-DNB product development without further increasing the dispersion. Stop reaction times of 0, 2, 4, and 5 min were assessed with a maximum increase in absorbance after 4 min of stopped time in the reaction coil (Figure 3).

The dispersion of the aliquots is essential for the partial zones overlap and consequent reaction. Also, the aspiration order is very important since the implemented sequence must ensure contact between the enzyme, the substrate, and the cofactor to maximise the chemical reaction. Hence, the aspiration order of the aliquots was also studied. The aspiration order CDNB – DMSO/inhibitor – GST – GSH was selected because the analytical signal is 4.3 times higher than the aspiration order CDNB – GST – DMSO/inhibitor – GSH and 1.7 times higher than the aspiration order GSH – GST – DMSO/inhibitor – CDNB/GSH – DMSO/inhibitor – GST – CDNB. Previously reported GST P1 assays are conducted at either 25 or 37 °C^27^^,^^42^^,^^43^. To guarantee the best analytical signal and to simulate body temperature, 37 °C was used. Different GSH (12 mM) and GST P1(5 × 10^−6 ^g mL^−1^) volumes were also tested with 20 µL being optimal for both. The flow rate of the propulsion to the detector was studied between 1- and 2-ml min ^−1^. It is evident that using the higher flow rate (2 ml min ^−1^), we obtained a higher absorbance of the final product (increases 1.5 times).

Using the optimised parameters, the analytical characteristics of the system were determined, to afford the concentration range in which there is a linear relationship between the CDNB concentration and the spectrophotometric signal. A calibration curve was obtained using standard solutions of increasing concentrations of CDNB. The obtained calibration curve was Abs = (0.09 ± 0.02) C (mM) + (0.14 ± 0.02); *R*^2^ = 0.99, where Abs and C correspond to the absorbance intensity and the concentration of CDNB in mM, respectively, with a confidence limit for the intercept and slope of 95%. The linearity range of this method is between 0.85 and 44 mM. All the analytical features of this calibration curve are represented in Table 3.

**Table 3.** Analytical features of the calibration curve.

Analytical features	Values
Detection limit	0.26 mM
Quantification limit	0.85 mM
*R* ^2^	0.99
Slope	0.09
Intercept	0.14
Standard error of slope (Sb)	0.006
Standard error of intercept (Sa)	0.007
Standard error of regression	0.008
Sum of squares of the regression	0.01
Sum of squares of the residuals	0.0002

### 3.2. Determination of GST P1-1 inhibition by organometallic compounds

The optimised GST P1-1 SIA method was used to determine the inhibition profiles of a library of organometallic compounds. Each concentration of each compound was performed in triplicate using a 1.8 mM of CDNB solution which was defined from the linear concentration range of the calibration curve.

In Figure S1 in the Supplementary Material, it is represented the obtained polynomial relations depending on the normalised activity and each compound logarithm concentration. The resulting IC_50_ values of the compounds are given in Table 4. The RSD obtained for all the IC_50_ obtained with the new methods is around 7 (*n* = 20).

**Table 4.** GST P1-1 inhibition of ethacrynic acid and a series of organometallic compounds.

Compound	Structure^a^	IC_50_ (µM ± SD)
**1** Ethacrynic acid	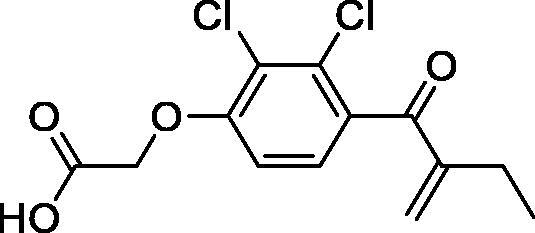	11.3 ± 0.8
**2a**	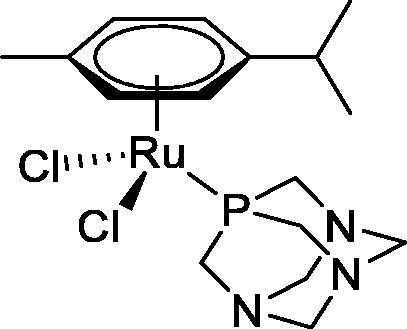	57 ± 4
**2b**	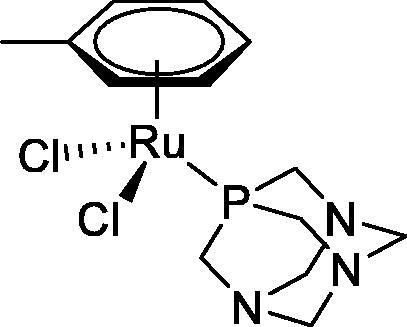	76 ± 6
**2c**	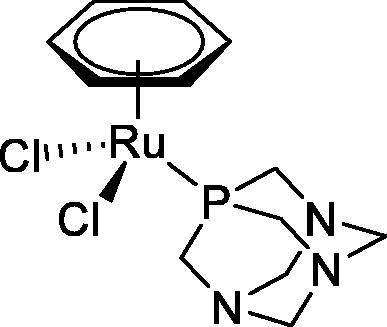	24.7 ± 1.4
**2d**	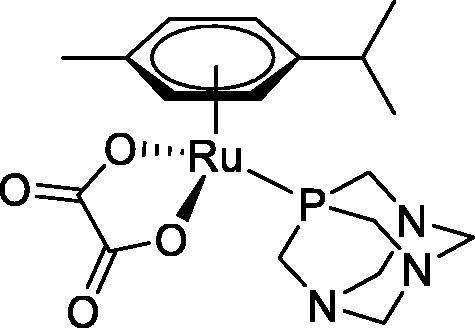	61 ± 5
**3a**	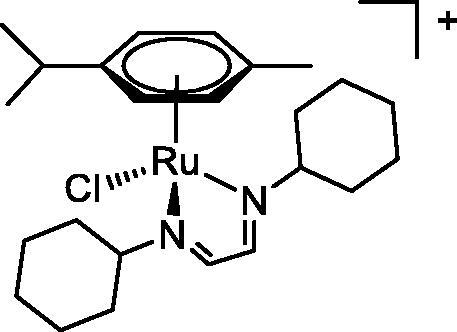	235 ± 2
**4a**	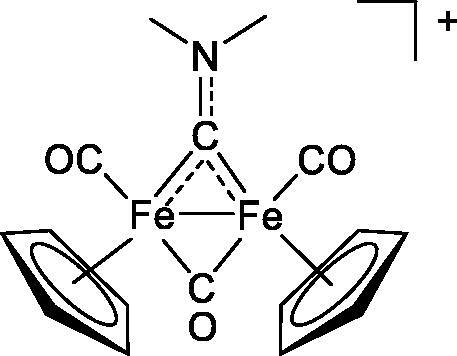	275 ± 9
**4b**	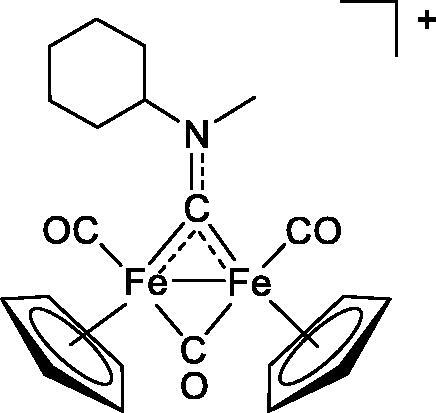	219 ± 8
**4c**	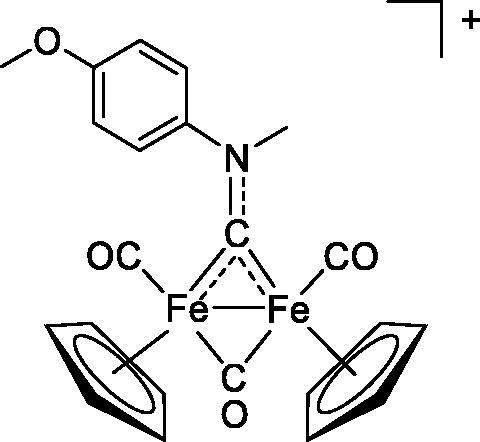	113 ± 5
**4d**	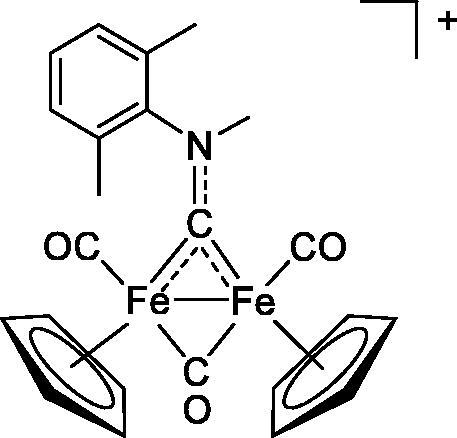	72 ± 5
**5a**	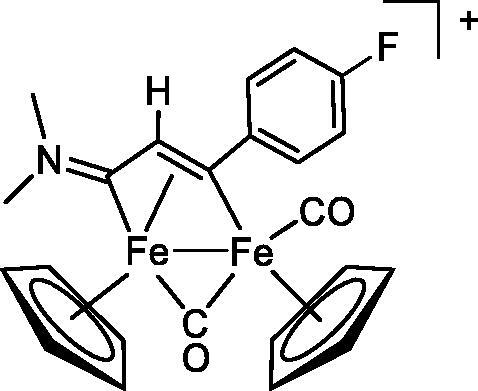	83 ± 9
**5b**	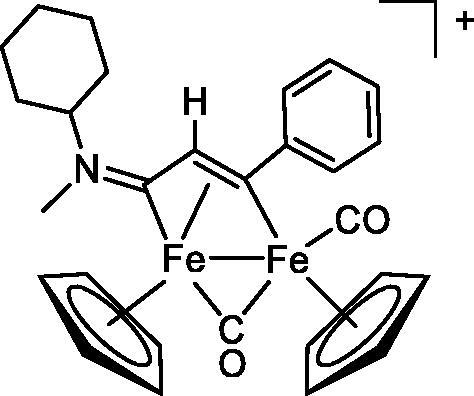	91 ± 9
**5c**	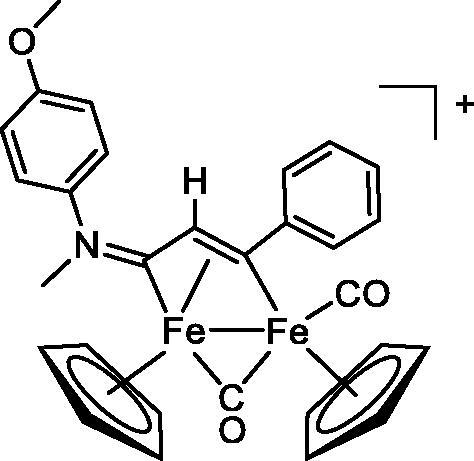	46.8 ± 2.8
**5d**	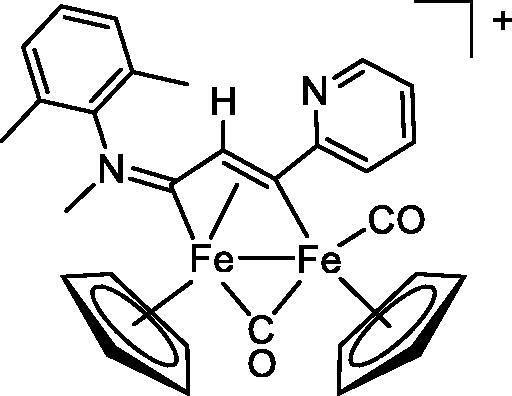	34.0 ± 4.3
**5e**	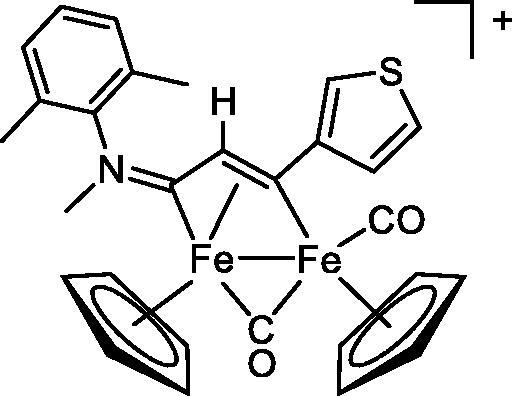	30.3 ± 8.3
**6a**	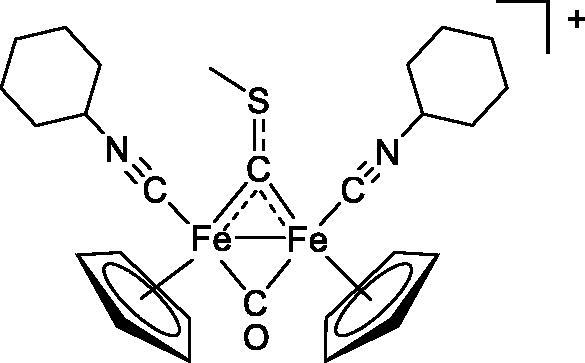	17.4 ± 2.8
**6b**	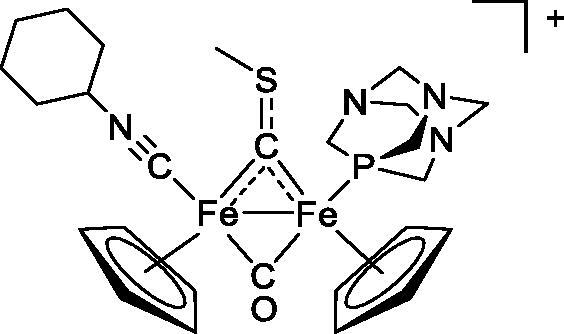	165 ± 4
**7a**	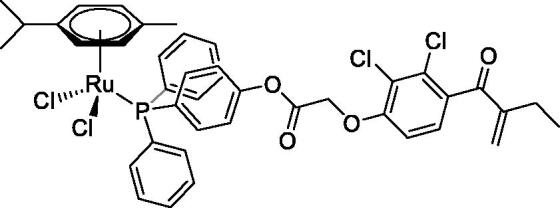	181 ± 7
**7b**	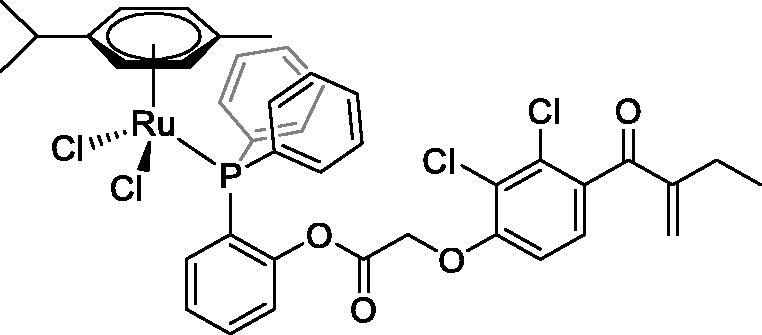	152 ± 6
**7c**	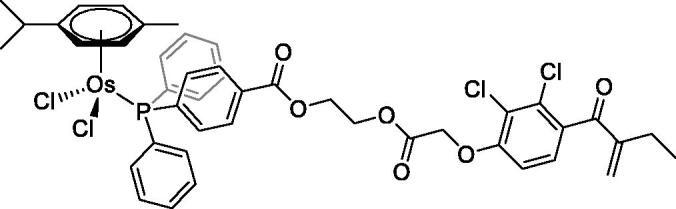	138 ± 12
**7d**	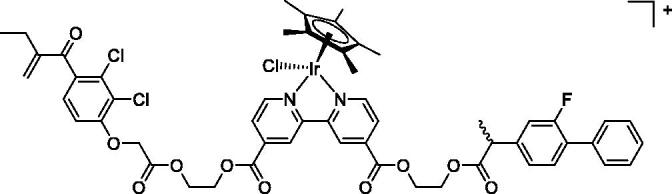	6.7 ± 0.7
**7e**	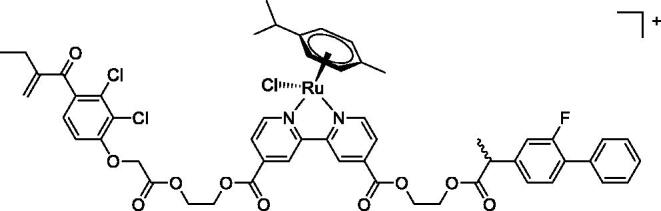	12.1 ± 1.8

^[a]^ Cationic diiron complexes as CF_3_SO_3_^−^ salts; cationic Ru/Ir complexes (**3a**, **7d**, **7e**) as NO_3_^−^ salt.

The known GST P1-1 inhibitor, ethacrynic acid (see Introduction), was used as a positive control. The literature reports different IC_50_ values for **EA** ranging from 4.9 µM^44^ to 12 µM^27^, the latter being close to the IC_50_ of 11.3 ± 0.8 µM obtained using the SIA system. The organometallic compounds display IC_50_ values ranging from 6.7 ± 0.7 to 275 ± 9 µM with the results allowing some structure-activity relationships to be ascertained. RAPTA complexes **2a–d** showed a modest GST P1-1 inhibitory activity (average IC_50_ 54 µM), albeit considerably higher than the related Ru(II)-arene compound **3a** (RUCYN, IC_50_ 235 µM). Conjugation of **EA** to the Ru(II) and Os(II) η^6^-arene complexes via a modified triaryl phosphine ligand (complexes **7a–c**) does not result in effective GST P1-1 inhibitors with IC_50_ in the range 137–181 µM. In this respect, modest GST inhibitory activity was previously ascertained for **7c** and related Os(II)-EA conjugates in ovarian cancer cell lines (A2780, A2780cisR) ^38^. In contrast, the Ru(II) (**7d**) and Ir(III) (**7e**) derivatives with a doubly-derivatized **EA** and flurbiprofen 2,2′-bipyridine ligand are potent GST P1 inhibitors. The iridium compound **7d** is the strongest inhibitor of GST P1 (IC_50_ = 6.7 ± 0.7 µM) in the present work, more effective than **EA**. Compound **7e** exhibits significant cytotoxicity on a panel of cancer cell lines, with its biological activity benefiting from the combined action of the metal scaffold and the two enzyme inhibitors^37^.

The diiron cyclopentadienyl complexes with aminocarbyne (**4a–d**) and vinyliminium (**5a–e**) ligands are either modest inhibitors of GST P1-1 or are essentially inactive, with IC_50_ values in the range of 30–275 µM. The presence of (hetero)aromatic substituents on the bridging ligand is correlated with an increase in inhibitory activity, e.g. compare the IC_50_ values of **4a,b,** and **5a,b** with **4c,d,** and **5c,d,e**. The thiocarbyne complex **6a**, with two cyclohexyl isocyanide ligands, is a comparatively good GST P1-1 inhibitor with an IC_50_ value of 17.4 ± 2.8 μM. Notably, the introduction of a PTA ligand in **6b** dramatically impairs the ability of the compound to inhibit GST P1-1. Nevertheless, both **6a** and **6b** are effective DHFR reductase inhibitors.^45^

### 2.3. Validation of the SIA system

To ensure the validation of the newly developed methodology, some compounds were also analysed using a batch procedure. The IC_50_ values obtained were compared with those obtained from the SIA method in Table 5 and are in reasonable agreement, showing the same trend and similar values for the active inhibitors.

**Table 5.** Assessment of SIA and batch IC_50_ values.

Compounds	IC_50_ batch ± SD (µM)	IC_50_ SIA ± SD (µM)
**1**	13.58 ± 0.02 ^(1)^	11.3 ± 0.8
**4a**	226 ± 3	275 ± 9
**4c**	61 ± 3	113 ± 5.3
**4d**	33 ± 6	72 ± 5
**6a**	11.5 ± 0.7	17 ± 3
**7e**	11.6 ± 0.5	12 ± 2

IC_50_ value obtained using the same batch method in reference^41^.

The results were evaluated using the *t*-test, carried out as a bilateral coupled test. In agreement with the student’s *t*-test, the tabulated *t* value (2.57), is lower than the calculated *t* value (2.3). Thus, there are no statistical differences at the confidence level of 95%^46^, further confirmed by a linear correlation as described by the equation:

(1) $${\mathrm{IC}}_{50}\text{ SIA }=(1.2\pm 0.3)\;{\mathrm{IC}}_{50}\;\mathrm{BATCH}-(12.4\;\pm \;30.9)$$

where IC_50_SIA and IC_50_BATCH are, respectively, the IC_50_ results acquired using the SIA and batch methods, with intercept and slope confidence limits of 95%. The predictable intercept and slope values were not considered significantly different, respectively, from 0 to 1, confirming the SIA and batch methods agreement. The coefficient of Pearson correlation for the two methods is near 1 (∼ 0.98).

According to the goal of this study and all the advantages of using the SIA methodology such as robustness, reproducibility, versatility, computer control, and reliability, the analytical signal is obtained in 5 min whereas 8 min are required for the batch procedure. The SIA system also requires fewer materials than in batch method, i.e. 5 times less GSH solution, 1.25 times less CDNB solution, and 2.3 times less GST P1-1 solution.

## 4. Conclusions

An SIA system was developed to evaluate the GST P1-1 inhibition capacity of organometallic complexes with putative anticancer activity. Some of the compounds tested exhibited good inhibition profiles with the low µM range of IC_50_ values and were comparable to the benchmark organic inhibitor, EA. It is therefore expected that these compounds could be useful to treat cancers where GST P1-1 is overexpressed^47–49^. The SIA method was found to be a good alternative to the batch method reducing the analysis time and the number of reagents required. Hence, the SIA method is considered an important automatic alternative for the analysis of GST P1-1 inhibitors.

## Supplementary Material

Supplemental Material
