# Supplementary material for: Automated approach for the evaluation of glutathione-S-transferase P1-1 inhibition by organometallic anticancer compounds
Source: J Enzyme Inhib Med Chem. 2022 May 29;37(1):1527–36. doi: 10.1080/14756366.2022.2073443 (PMC9176637; doi:10.1080/14756366.2022.2073443)
Supplement: Supplemental Material [file IENZ_A_2073443_SM8477.pdf]

Figure S1 - Experimental inhibition data of the compounds tested in the GST P1-1 inhibition assays.

4a

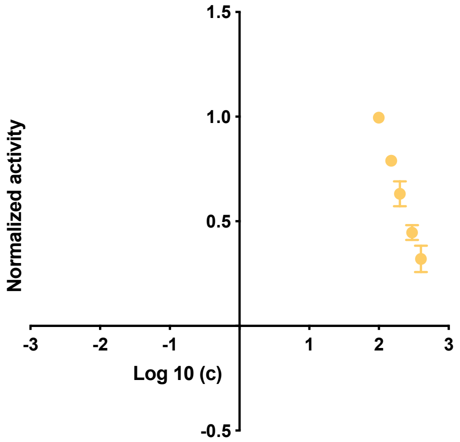

4b

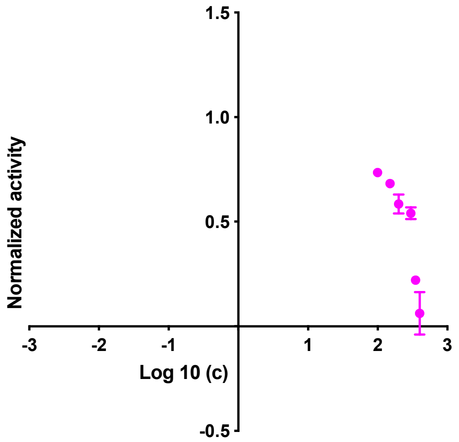

4c

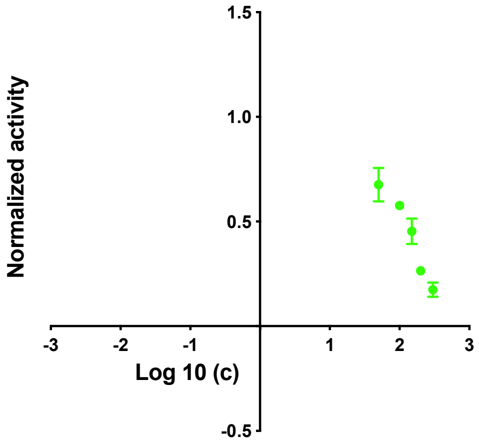

4d

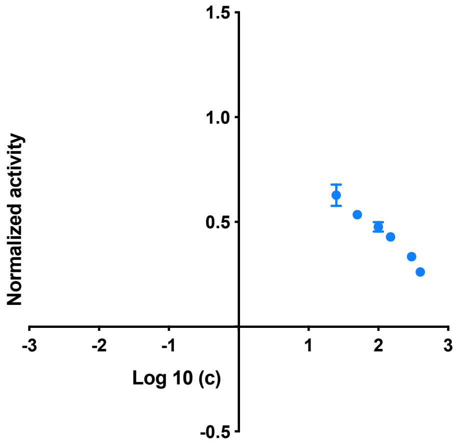

5a

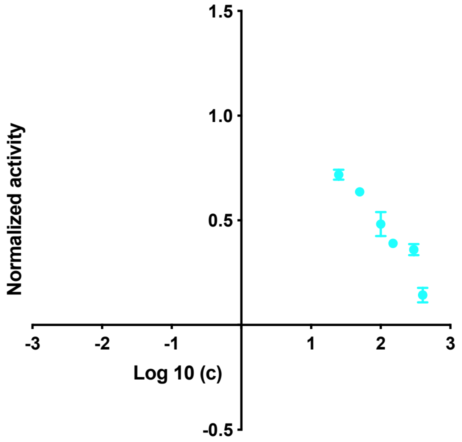

5b

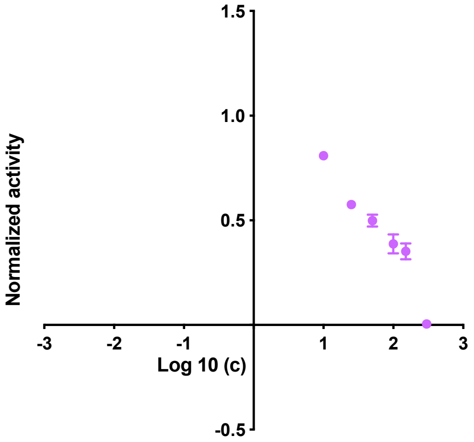

5c

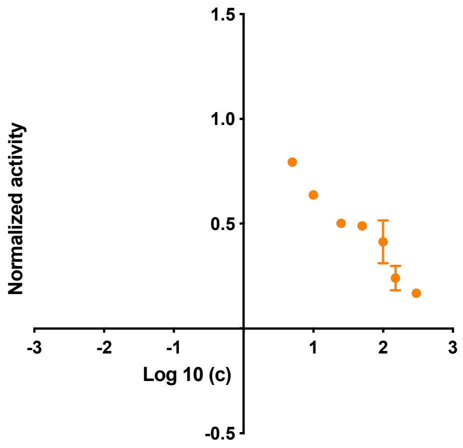

5d

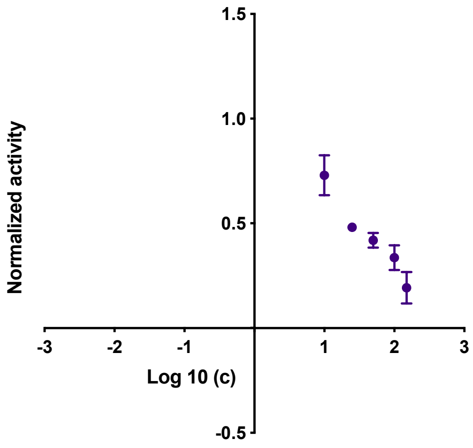

5e

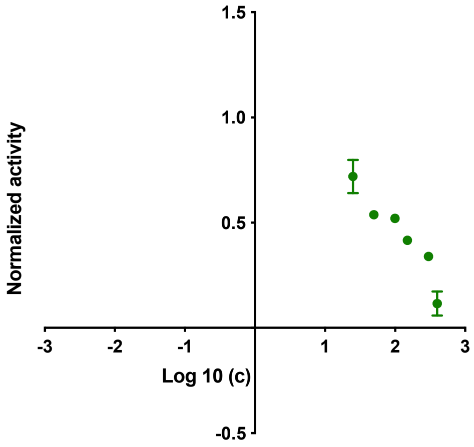

6a

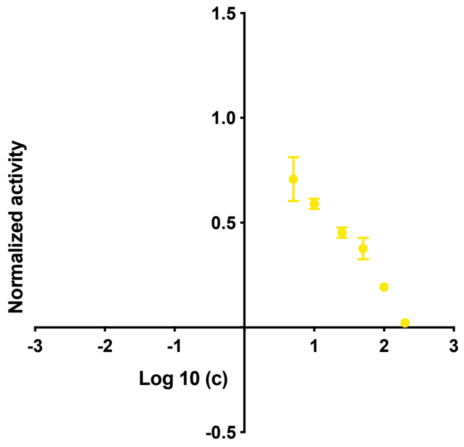

6b

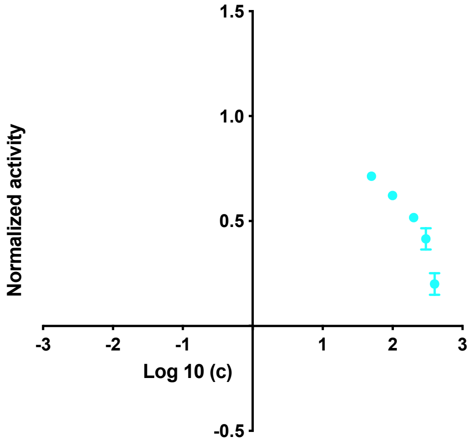

7a

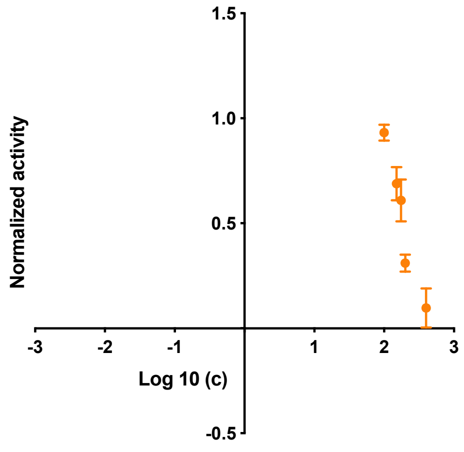

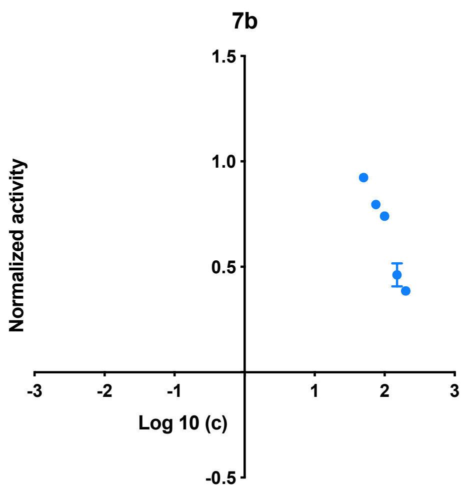
7b
Normalized activity
Log 10 (c)
1.5
1.0
0.5
-0.5
-3
-2
-1
1
2
3

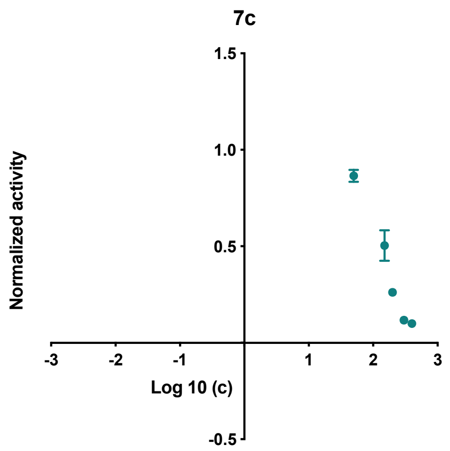
7c
Normalized activity
Log 10 (c)
1.5
1.0
0.5
-0.5
-3
-2
-1
1
2
3

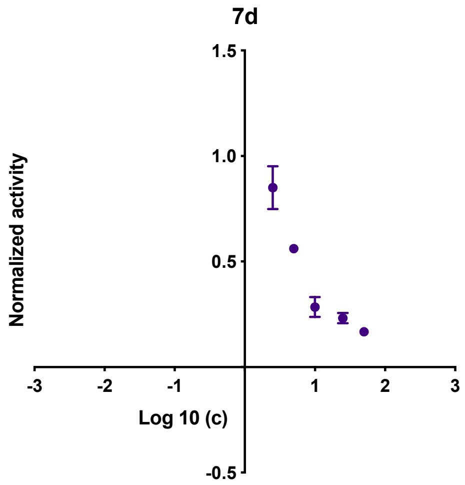
7d
Normalized activity
Log 10 (c)
1.5
1.0
0.5
-0.5
-3
-2
-1
1
2
3

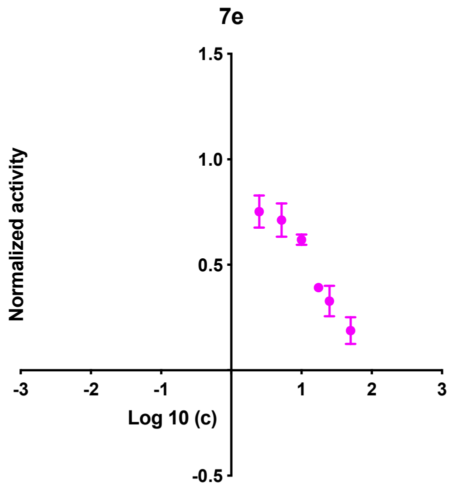
7e
Normalized activity
Log 10 (c)
1.5
1.0
0.5
-0.5
-3
-2
-1
1
2
3

Table S1 – Fitting parameters of each curve shown in Fig S1.

**Compound**	**Coefficient of determination R^2**
**1 Ethacrynic acid**	0.98
**2a**	0.98
**2b**	0.96
**2c**	0.99
**2d**	0.97
**3a**	0.95
**4a**	0.99
**4b**	0.78
**4c**	0.94
**4d**	0.98
**5a**	0.90
**5b**	0.93
**5c**	0.93
**5d**	0.95
**5e**	0.96
**6a**	0.98
**6b**	0.88
**7a**	0.96
**7b**	0.92
**7c**	0.97
**7d**	0.87
**7e**	0.94
